# A novel oral camptothecin analog, gimatecan, exhibits superior antitumor efficacy than irinotecan toward esophageal squamous cell carcinoma in vitro and in vivo

**DOI:** 10.1038/s41419-018-0700-0

**Published:** 2018-05-31

**Authors:** Jianling Zou, Shuang Li, Zuhua Chen, Zhihao Lu, Jing Gao, Jianyin Zou, Xiaoting Lin, Yanyan Li, Cheng Zhang, Lin Shen

**Affiliations:** 1 Department of Gastrointestinal Oncology, Key laboratory of Carcinogenesis and Translational Research (Ministry of Education), Peking University Cancer Hospital & Institute, Beijing, China; 2 Department of Otolaryngology, Otolaryngology Institute of Shanghai Jiao Tong University, Shanghai Jiao Tong University Affiliated Sixth People’s Hospital, Shanghai, China

## Abstract

Esophageal squamous cell carcinoma (ESCC) is a frequently diagnosed and deadly malignancy with few standard therapeutic options. Camptothecins are considered one of the most promising antitumor drugs. A modified lipophilic analog, gimatecan, was synthesized as a novel oral camptothecin and showed impressive effects in various tumors, but its therapeutic efficacy and mechanisms in ESCC remain unclear. This study investigated the antitumor efficacy and mechanisms of gimatecan in ECSS both in vitro and in vivo. Using ESCC cell lines, cell line-derived xenografts and patient-derived xenografts models, we evaluated gimatecan’s inhibition of tumor growth, and compared its antitumor efficacy with that of irinotecan. Topoisomerase I function and expression were assessed using the DNA relaxation assay and Western blotting, respectively. DNA damage was evaluated by Western blotting. Cell cycle progression and cell apoptosis were assessed using flow cytometry and Western blotting. Gimatecan could significantly suppress tumor growth in vivo and inhibit tumor cell proliferation in vitro, which was superior to irinotecan. Gimatecan suppressed the function and expression of topoisomerase I. It also caused DNA damage and activated the phosphorylation of multiple checkpoint gatekeepers, such as ATM, ATR, BRCA1, H2AX, CHK1, CHK2, and p53. It induced S phase arrest, enhanced the expression of p21^WAF1/CIP^, and suppressed the expression of CDK2 and cyclin A. Induction of apoptosis was accompanied by increases in Bax, cleaved-caspase 3 activation, cleaved-caspase 9 induction, and a decrease in Bcl-2. The molecular and phenotypic changes induced by gimatecan were stronger than that of irinotecan. In ESCC, gimatecan suppressed the expression and function of topoisomerase I, induced DNA damage and intra-S phase cell cycle arrest, and resulted in apoptosis. And the results suggest that gimatecan has higher potency in inhibiting ESCC tumor growth than irinotecan, providing a rational novel therapeutic strategy for future clinical evaluation.

## Introduction

Esophageal cancer (EC) is the fourth most commonly diagnosed and the most fatal cancer in China^1^. Esophageal squamous cell carcinoma (ESCC) is the predominant histological type of EC, comprising more than 95% of all EC cases^2^. ESCC is considered an aggressive malignancy due to the poor prognosis and high mortality rate. Most patients that are diagnosed with locally advanced or metastatic ESCC at the time of initial diagnosis^3,4^ are unable to undergo radical surgery, so the mainstays of treatment for these patients are radiation therapy and chemotherapy. However, the prognosis for patients with ESCC is still poor, with a 5-year survival rate of only about 20%^3^. Therefore, it is crucial to identify the novel therapeutic alternatives or agents for patients with ESCC.

Due to their ability to disturb the catalytic cycle of DNA topoisomerase I, camptothecins are among the most promising antitumor drugs. They stabilize the covalent enzyme–DNA complex (cleavable complex) by forming a drug–enzyme–DNA complex during DNA synthesis, which is non-lethal and reversible. However, this causes the formation of irreversible double-stranded DNA breaks when a DNA replication fork collides with the cleavable complex^5,6^, Camptothecins can show preferential or selective toxicity to proliferating cells, particularly tumor cells, which are highly proliferative. As a result, the camptothecin analogs topotecan and irinotecan have already been widely used to treat several solid tumors, including colorectal carcinoma^7,8^ and lung cancer^9–12^. Recently, irinotecan has also shown promising results for the treatment of advanced ESCC^13–15^.

Although existing camptothecins have shown good tolerance and activity, a low therapeutic index is still the main disadvantage of clinical applications, which is largely attributed to the lability of the drug–enzyme–DNA complex and instability of the lactone ring. Consequently, research has aimed to enhance the antitumor efficacy, including modification of the molecular structure to induce topoisomerase I-mediated DNA cleavage and/or to stabilize the drug–enzyme–DNA complex^16,17^. The novel modified lipophilic analog gimatecan has been developed on the basis of this rationale, with the substitution at position C-7 by lipophilic chains^18,19^. This modification enhances rapid agent intake and stable drug interactions with intracellular targets^20,21^, and also allows the oral administration of gimatecan, which has shown advantages over oral topotecan in terms of the antitumor efficacy and therapeutic index in preclinical studies of non-small lung cancer and colon carcinoma^22,23^.

However, there has been no research into the application of gimatecan for ESCC, and the mechanism through which gimatecan suppresses proliferation of tumors remains unclear. This research assessed the antitumor efficacy of gimatecan and investigated its mechanism in ESCC.

## Results

### Gimatecan inhibits tumor proliferation of ESCC in vivo

**Fig. 1:**
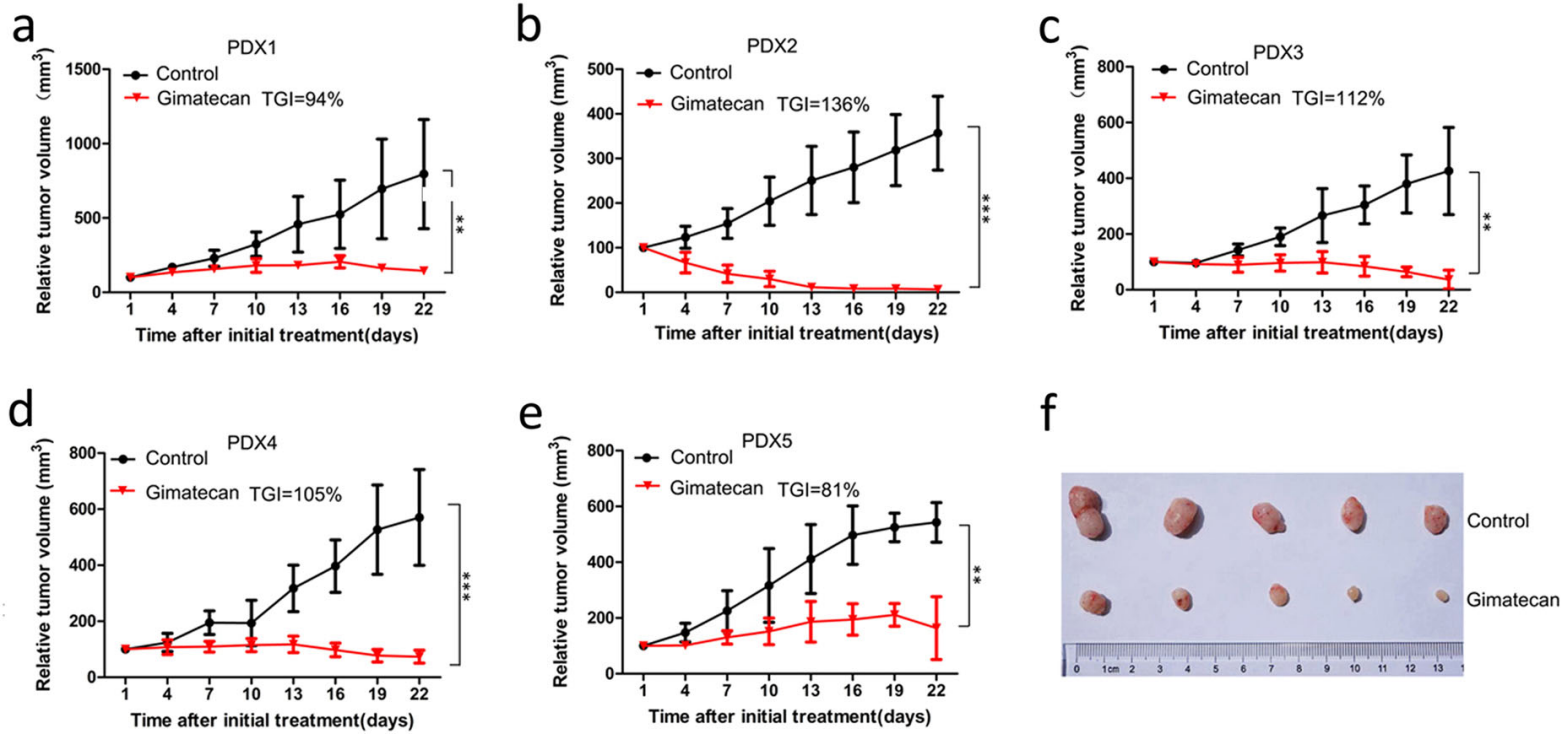
Gimatecan inhibits tumor growth in patient-derived xenograft (PDX) models of esophageal squamous cell carcinoma (ESCC). **a**–**e** In vivo antitumor activity of gimatecan in ESCC PDX models. Tumors were subcutaneously engrafted and grown in NOD/SCID mice until they were 150–200 mm^3^ in size. Then the mice were treated with saline containing 10% DMSO or gimatecan (0.25 mg/kg, d1–d5/week, oral gavage) for 3 weeks, and tumors were measured twice a week. Tumor volume is expressed as the mean ± SD of at least five mice in each group. Antitumor activity was analyzed using unpaired two-tailed *t-*tests, and is depicted by tumor growth inhibition. **P < 0.01, ***P < 0.001, **f** Images of tumors dissected out from killed mice. A representative image of PDX 2 at the end of treatment is shown

To evaluate the antitumor activity of gimatecan in vivo, five cases of PDX models were selected and treated with saline containing 10% DMSO (control) or gimatecan for 3 weeks. Compared to the control group, tumor growth was significantly suppressed in gimatecan-treated groups (TGIs were 94%, 136%, 112%, 105%, and 81% in five cases, all *p* < 0.01) (Fig. 1a–e). A representative image of PDX1’s tumor at the end of treatment is shown in Fig. 1f.

### Gimatecan shows antitumor effects that are superior to irinotecan

**Fig. 2:**
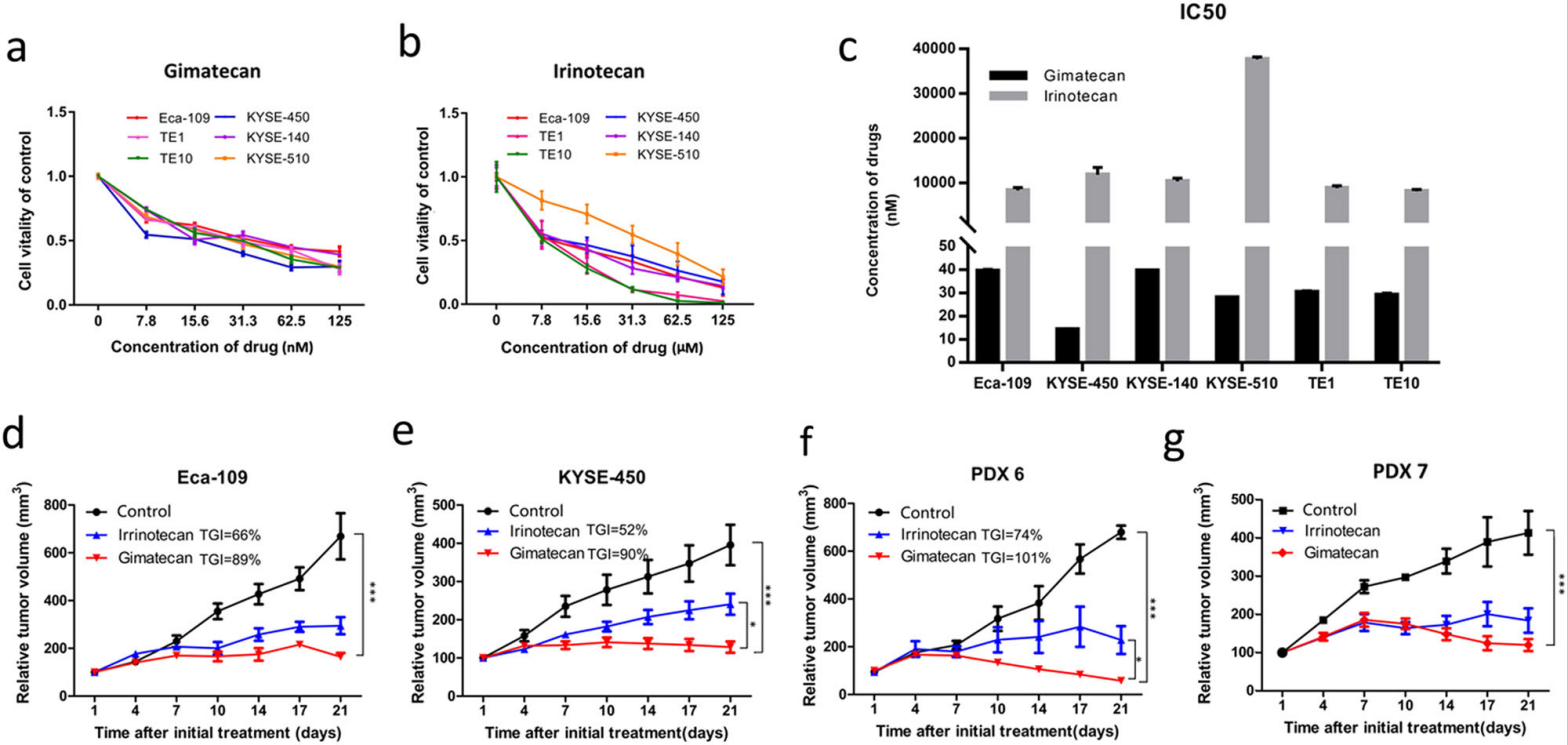
Gimatecan exhibits superior antitumor effect than irinotecan in vitro and in vivo. **a**, **b** Eca-109, KYSE-450, KYSE-140, KYSE-510, TE1, TE10 cells were seeded in 96-well plates overnight in complete medium and then exposed to a gradient dilution of gimatecan (0–125 nM) or irinotecan (0–125 μM) for 48 h. Cell viability was measured, and is presented as the mean ± SD of six replicate assays. **c** The IC50 values of gimatecan and irinotecan for each cell lines were calculated. Data are presented as the mean ± SD of three replicate assays. **d**–**g** In vivo antitumor activity of gimatecan and irinotecan in xenograft models of ESCC cell lines and patient-derived xenograft (PDX) models. Eca-109/KYSE-450 cell lines or PDX tumor tissues were subcutaneously engrafted and grown in NOD/SCID mice until 150–200 mm^3^. Then the mice were treated with saline containing 10% DMSO, gimatecan (0.25 mg/kg, d1–d5/week, oral gavage), or irinotecan (8 mg/kg, twice a week, i.p.) for 3 weeks, and tumor volume and the body weight of each mouse were measured twice a week. Tumor volume is expressed as the mean ± SD of at least five mice in each group. Antitumor activity was analyzed using an unpaired two-tailed *t-*test and is depicted by tumor growth inhibition. *P < 0.05, **P < 0.01, ***P < 0.001

The capacity of gimatecan to inhibit cell proliferation was assessed in ESCC cell lines EC-109, KYSE450, KYSE-140, KYSE-510, TE-1, and TE-10 with gradient dilutions for 48 h, compared with irinotecan. Gimatecan showed strong inhibition in a dose-dependent manner at nanomolar concentration (Fig. 2a), which was lower than that of irinotecan (micromole level, Fig. 2b). Similarly, the IC50 of gimatecan in all chosen cell lines ranged from 4.9 ± 0.47 nM to 39.6 ± 0.32 nM, which were lower than that of irinotecan (8140 ± 366–37,680 ± 521 nM), demonstrating that gimatecan inhibited cell proliferation of ESCC more effectively than irinotecan (Fig. 2c).

We then compared the antitumor effects of gimatecan to those of irinotecan in vivo. ESCC xenografts derived from cell lines Eca-109/KYSE-450 as well as two PDX models were used to evaluate the antitumor effects of gimatecan and irinotecan in vivo (Fig. 2d–g). The results showed that gimatecan had a superior antitumor effect than irinotecan, particularly in the KYSE-450 xenograft (TGI = 90% vs. 52%, *p* < 0.05) and PDX6 (TGI = 101% vs. 74%, *p* < 0.05). The Eca-109 xenograft (TGI = 89% vs. 66%, *p* = 0.06) and PDX7 (TGI = 94% vs. 73%, *p* = 0.08) also showed greater inhibition of the tumor. Moreover, the given dosage did not cause significant loss of body weight.

### Gimatecan reduces topoisomerase I specific activity

**Fig. 3:**
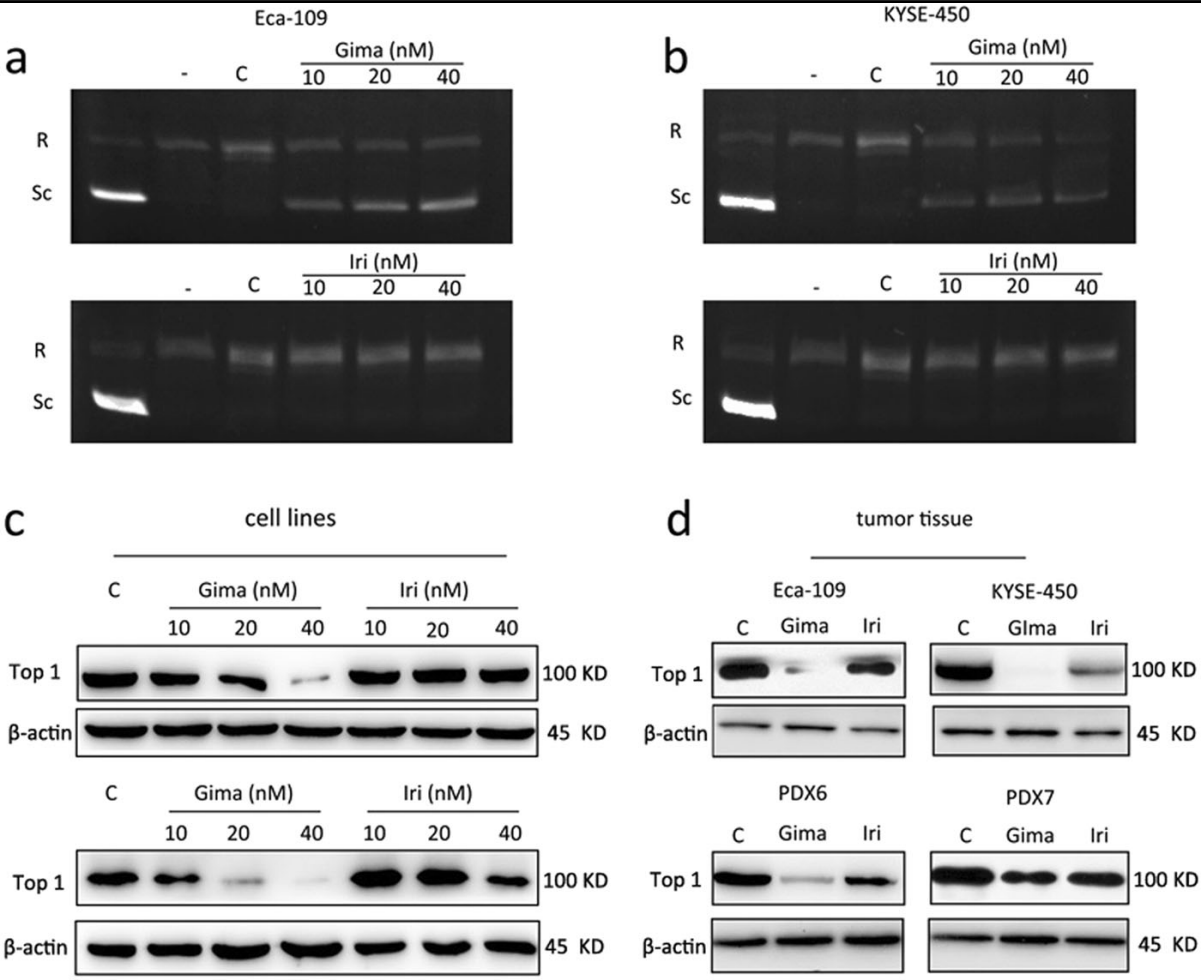
Gimatecan reduces topoisomerase I specific activity and suppress topoisomerase I expression in ESCC. **a**, **b** Experiment of topoisomerase I activity using different assay: Eca-109 and KYSE-450 cell lines were exposed to serial dilutions (10 nM, 20 nM, and 40 nM) of gimatecan and irinotecan for 2 h, and 20 µL of reaction containing nucleoli extract protein was incubated with supercoiled DNA for 30 min. Lane 1, supercoiled DNA only. Lane 2, relaxed DNA used as negative control. Lanes 3–5, supercoiled DNA, and nucleoli extract protein of cells treated with different concentration of gimatecan or irinotecan for 2 h; **c**, **d** the expression of topoisomerase 1 was assessed by Western blotting in vitro and in vivo. Eca-109 and KYSE-450 cell lines were exposed to 10 nM, 20 nM, and 40 nM gimatecan or irinotecan for 48 h, and harvested at 70–80% confluence. For in vivo experiment, the mice were killed and tumor tissues of Eca-109 and KYSE-450 cell line xenografts and PDX models were harvested at the end of treatment. Total protein was extracted from harvested cell lines or tumor tissues, and the expression of topoisomerase 1 was assessed by Western blotting

To explore the mechanisms of gimatecan, we first studied its inhibition of topoisomerase I using the DNA relaxation assay, compared with irinotecan. Topoisomerase I specific activity was inhibited by gimatecan in Eca-109 and KYSE-450 cells at 10 nM, 20 nM, and 40 nM (Fig. 3a, b). Untreated cells had dominant proportions of relaxed DNA, while gimatecan-treated cells displayed a high ratio of the supercoiled form, indicating the loss of topoisomerase I activity. However, irinotecan treated cells at the same concentration mainly displayed relaxed DNA form. To verify the mechanisms, we increased the dosage of irinotecan, and found that irinotecan could also inhibit topoisomerase I specific activity in a dose- and time-dependent manner in both cell lines (Supplement Fig. 1a, b). Such as, when treated with 40 μM irinotecan for 2 h, almost 100% of cellular DNA remained supercoiled, which was similar with the effect of 80 nM gimatecan (Supplement Fig. 1a). A similar phenomenon was seen when cells were treated with 30 nM gimatecan and 10 μM irinotecan for different periods of time (Supplement Fig. 1b). Apart from the function of topoisomerase I, protein expression was also explored by Western blotting, and the results showed that gimatecan more significantly decreased the topoisomerase I expression in Eca-109 and KYSE-450 cell lines than irinotecan (Fig. 3c), as well as in the tumor tissues (Fig. 3d). And the inhibition effect of gimatecan was stronger than irinotecan both in vitro and in vivo, which was consistent with our findings of ESCC proliferation and tumorigenesis.

### Gimatecan induces severe DNA damage

**Fig. 4:**
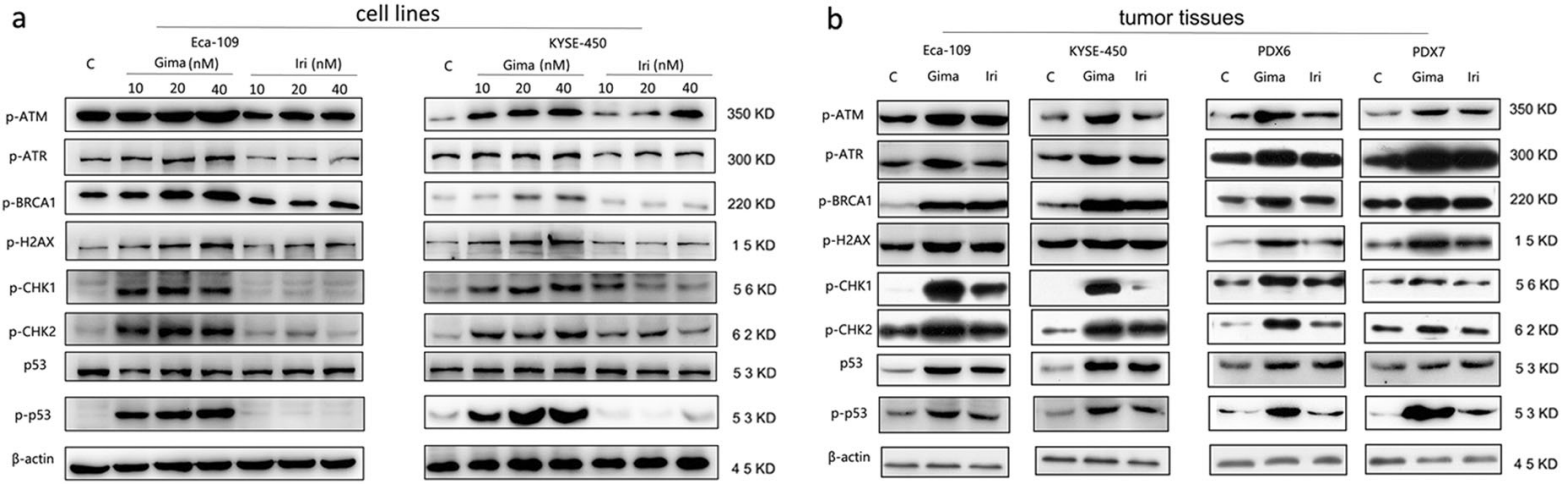
Gimatecan induces DNA damage in ESCC. The expression of DNA damage-related proteins was assessed by Western blotting (**a**) in vitro and (**b**) in vivo. Eca-109 and KYSE-450 cell lines were exposed to different concentration (10 nM, 20 nM, and 40 nM) of gimatecan and irinotecan for 48 h, and harvested at 70–80% confluence. At the end of in vivo treatment, the mice were killed and tumor tissues of Eca-109 and KYSE-450 cell line xenograft and PDX models were harvested. Total protein was extracted from harvested cell lines or tumor tissues, and the expression of the following DNA damage-related proteins were assessed by Western blotting: p-ATM, p-ATR, p-BRCA1, p-H2AX, p-CHK1, p-CHK2, p53 and p-p53

To characterize the DNA damage response during gimatecan treatment, the activation status and expression levels of proteins involved in DNA damage checkpoint pathways were measured in Eca-109 and KYSE-450 cell lines in vitro (Fig. 4a) and xenografts in vivo (Fig. 4b). In the event of DNA damage, those molecules are phosphorylated and activate downstream factors, such as ATM and ATR, the sensors of DNA damage; γ-H2AX and BRCA1, the mediators of DNA damage; Chk1 and Chk2, the transducers of DNA damage; and p53, the effector of DNA damage. The results showed that gimatecan treatment increased the expression of p-ATM, p-ATR, p-BRCA1, p-H2AX, and p-p53, indicating that gimatecan could activated the DNA damage pathway at 10 nM concentration, and the effect was stronger with the increase of gimatecan dosage to 20 nM and 40 nM. However, irinotecan did not induced significant effect at the same concentration. When we increased the concentration of irinotecan to 10 μM, similar changes were seen (Supplement Fig. 2). The results suggested that, both gimatecan and irinotecan could induced DNA damage, and the effects of gimatecan were more significant than those of irinotecan.

### Gimatecan induces S-phase arrest and apoptosis

**Fig. 5:**
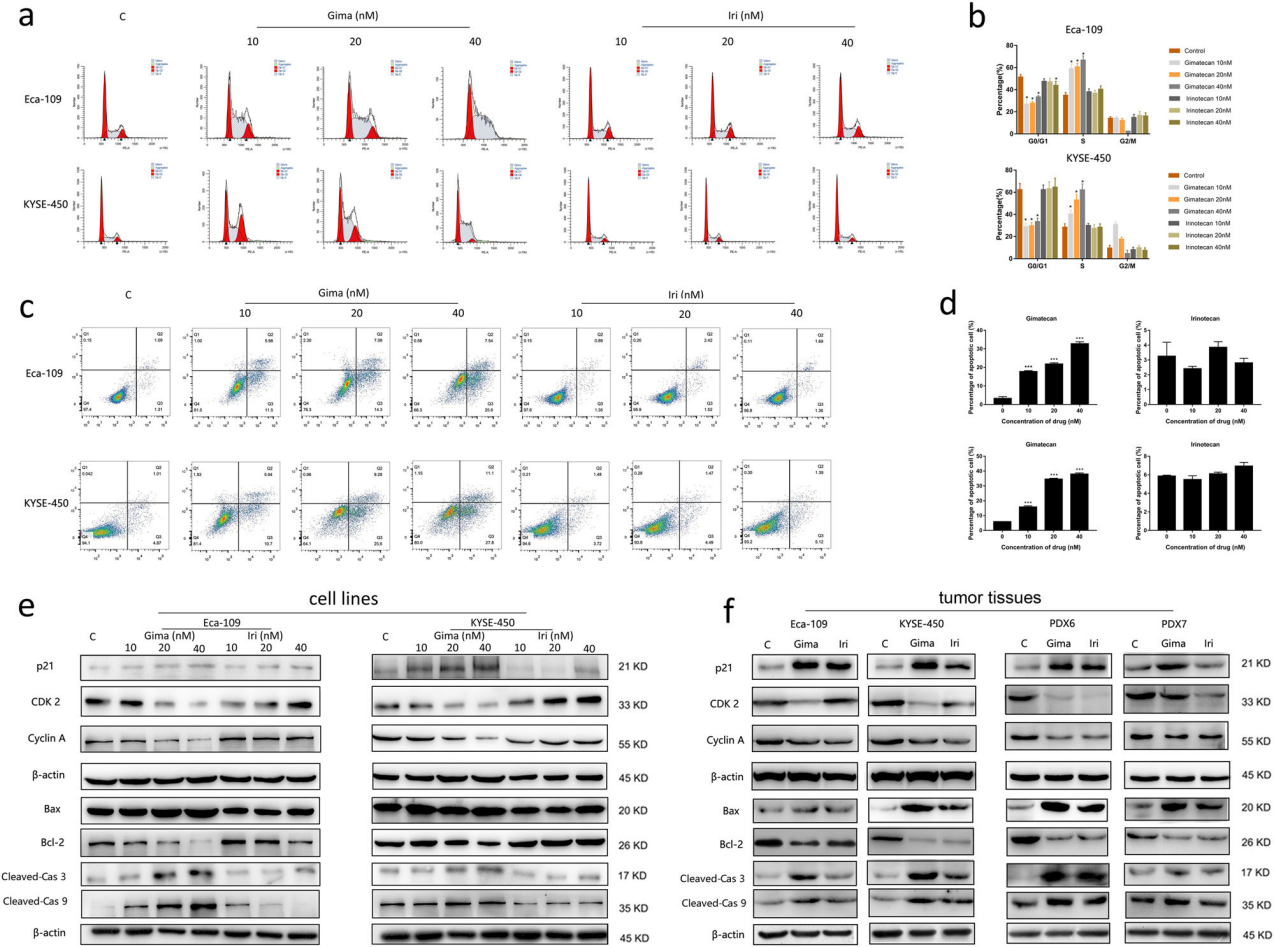
Gimatecan induces S-phase arrest and apoptosis in ESCC. **a** Eca-109 and KYSE-450 cells were treated with different concentration (10 nM, 20 nM, and 30 nM) of gimatecan and irinotecan for 4 h. Cell cycle progression was assessed using propidium iodide staining detected by fluorescence activated cell sorting. **b** Sums of percentages of each cycle were also calculated in Eca-109 and KYSE-450. Results are representative of three independent experiments. **c** Eca-109 and KYSE-450 cells were treated with gimatecan and irinotecan at the indicated dose for 72 h and stained with Annexin V-PE/7-AAD. **d** Sums of percentages of early apoptosis (Q3) and late apoptosis (Q2) were calculated as total apoptosis ratios. Results are representative of three independent experiments. **e**, **f** The expressions of proteins related to the cell cycle and apoptosis were assessed by Western blotting in vitro and in vivo. Eca-109 and KYSE-450 cell lines were exposed to 10 nM, 20 nM, and 40 nM concentration of gimatecan and irinotecan for 48 h, and harvested at 70–80% confluence. At the end of in vivo treatment, the mice were sacrificed and tumor tissues of Eca-109 and KYSE-450 cell line xenograft and PDX models were harvested. Cell cycle-related proteins, such as Cyclin A, CDK2, and p21, and Pro- and anti-apoptotic proteins including Bax, Bcl-2, cleaved-caspase 3, and cleaved-caspase 9 were assessed by Western blot. Data represent the mean ± SD of three replicate assays. *p < 0.05, **p < 0.01, ***p < 0.001

To detect how DNA damage affects cell cycle progression, we conducted cell cycle analyses in Eca-109 and KYSE-450 cell lines (Fig. 5a, b). First, we carried out propidium iodide staining to identify the cell cycle status after gimatecan and ironotecan treatment. Treatment with 10 nM, 20 nM, and 30 nM gimatecan resulted in 58.82% ± 4.58%, 60.46% ± 3.44%, 66.65% ± 3.86% of cells in the S-phase compared with the control group of 34.75% ± 2.54 % in Eca-109 (all *P* < 0.001). Similarly, treatment with 10 nM, 20 nM, and 30 nM gimatecan resulted in 40.28% ± 3.87%, 52.88% ± 4.97%, and 62.13% ± 5.34 % of cells in the S-phase compared with the control group of 28.33% ± 3.23 % in KYSE-450 cell lines after 4 h (all *P* < 0.001). However, the same concentration of irinotecan did not induced this effect (Fig. 5a). Histograms of each phase were shown in Fig. 5b. While when we increased the dosage of irinotecan to 10 μM, similar S-phase arrest induced by 30 nM gimatecan was seen at 4 h and 8 h after treatment (Supplement Fig. 3a).

To evaluate the effects of gimatecan on cell apoptosis, we performed apoptosis analysis in Eca-109 and KYSE-450 cell lines using Annexin V-PE/7-AAD staining (Fig. 5c, d). 10 nM, 20 nM, and 30 nM gimatecan induced a significant increase in the ratio of apoptotic cells in Eca-109 cell lines (17.76% ± 0.49%, 21.83% ± 0.66%, 32.75 % ± 1.02%, vs 3.25% ± 0.94% in the control group, all *P* *<* 0.001). For KYSE-450 cells, 10 nM, 20 nM, and 30 nM gimatecan induced 15.77% ± 0.78%, 34.55% ± 0.58%, and 37.93% ± 0.84% apoptotic cells respectively, compared with 5.85% ± 0.1% apoptotic cells in control group (all *P* < 0.001). However, irinotecan at the same concentration did not induce significant apoptosis (Fig. 5c). Column diagram of apoptotic cells were shown in Fig. 5d. While when we increased the dosage of irinotecan to 10 μM, significant cell apoptosis which was similar to that caused by 30 nM gimatecan could also be seen (Supplement Fig. 3b).

To further investigate the changes in cell cycle arrest and apoptosis, the expressions of proteins related to the cell cycle, and a series of anti-apoptotic and pro-apoptotic proteins were analyzed by Western blotting. The expression of p21 was upregulated, while the expression of cyclin A and CDK2 were downregulated after treating the Eca-109 and KYSE-450 cell lines with gimatecan. Moreover, the expression of Bcl-2, a key regulator of the mitochondrial membrane, was downregulated, while the expression of pro-apoptotic protein Bax significantly increased after gimatecan treatment. In addition, exposure to gimatecan enhanced caspase-dependent apoptosis and activated cleaved-caspase 3 and cleaved-caspase 9. Changes induced by gimatecan were stronger than those induced by the same concentration of irinotecan (Fig. 5e). When the concentration of irinotecan was increased to 10 μM, the similar changes of protein expression could also be seen (Supplement Fig. 3c). Moreover, the tumor tissues of the cell line xenografts and PDX models after gimatecan and irinotecan treatment exhibited similar results of S-phase arrest and apoptosis (Fig. 5f).

Taken together, these data clearly indicate that gimatecan induce S-phase arrest, and then activate apoptosis through the pro-apoptotic signaling pathway. More importantly, gimatecan induced more obvious changes in cell cycle arrest and apoptosis, which was also in accordance with the suppression of tumor growth in vivo.

**Fig. 6:**
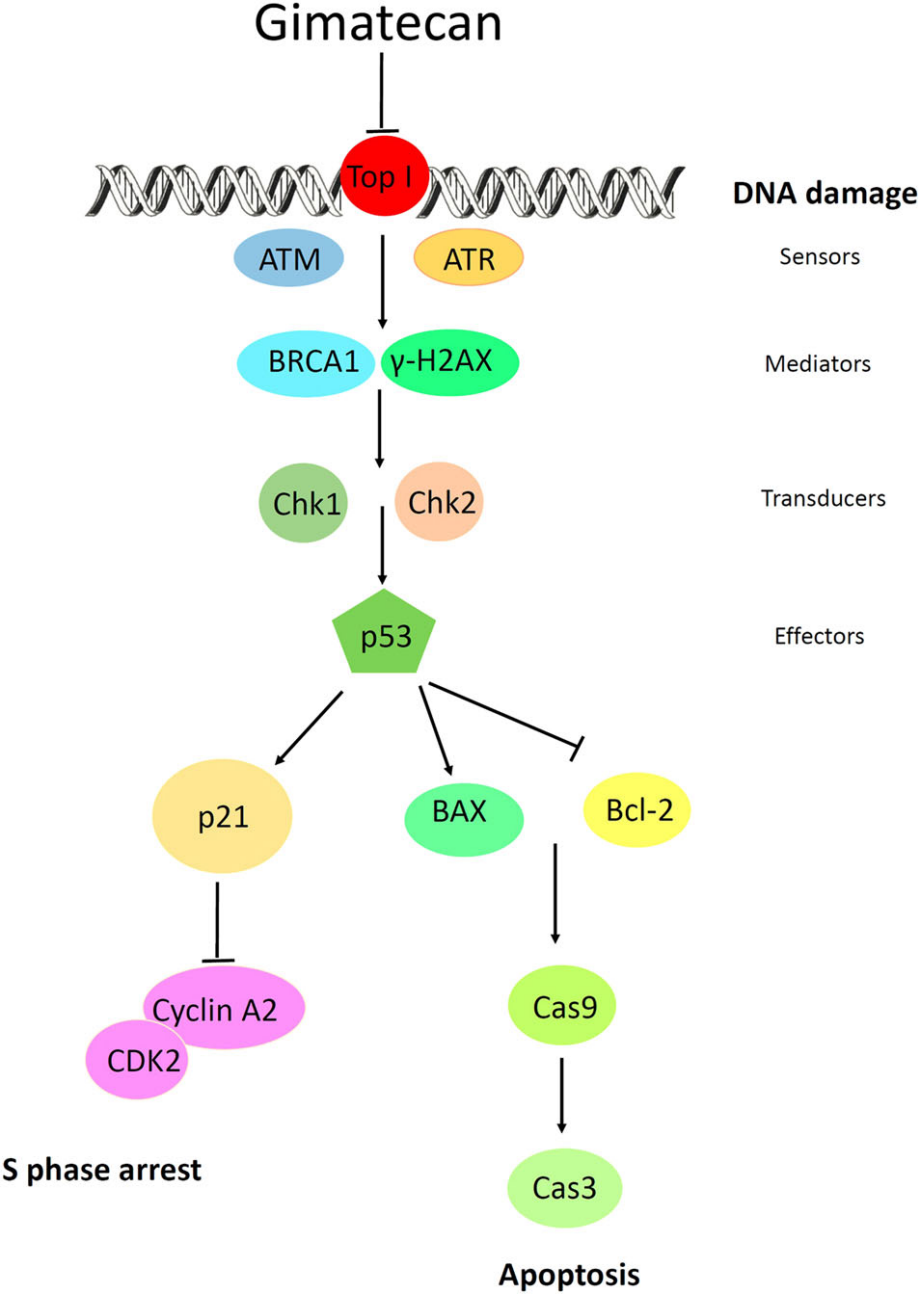
Proposed mechanisms of gimatecan-induced DNA damage, S-phase arrest, and apoptosis in ESCC

In summary, our data demonstrate that the anticancer activity of gimatecan is mediated through topoisomerase inhibition and subsequent induction of DNA damage. Then DNA damage response signaling activates p53 and leads to the accumulation of p21 and S-phase cell cycle arrest and induction of apoptosis in ESCC, as depicted and summarized in Fig. 6. The mechanism of gimatecan is similar to irinotecan, but gimatecan induces stronger effects.

## Discussion

We evaluated the chemotherapeutic effects and mechanisms of action of gimatecan, a lipophilic oral camptothecin analog, by comparing it to a first-line clinical agent, irinotecan. Gimatecan inhibited the proliferation of multiple EC cell lines, suppresses topoisomerase I activity, induced DNA damage, arrest cell cycle, caused cell apoptosis, and repressed tumor growth in mice models more significantly than irinotecan, demonstrating that it might potentially be a more powerful anticancer agent than irinotecan.

Exposure of cells to topoisomerase inhibitors could stabilize the cleavable complex. The appearance of collision between stable topoisomerase I-drug-DNA complexes and replication forks or transcription complexes generally induces lethal double-strand breaks in DNA^24^. The DNA breaks lead to DNA damage, which is the central mechanism of antitumor activity^25,26^. DNA damage triggers activation of DNA damage-response elements, such as ATM and ATR. We found that treatment with gimatecan induces phosphorylation of both ATM and ATR proteins in ESCC both in vitro and in vivo. Activated ATM and ATR, either directly or through sequential steps, phosphorylate the downstream proteins BRCA1, H2AX, Chk1, and Chk2, and subsequently affect downstream factors involved in cell cycle progression and cell survival^27–29^. The tumor suppressor protein p53 is a crucial component of cellular machinery that regulates various signaling pathways including DNA damage, the cell cycle, and apoptosis^30–32^. Under stressed conditions, such as induction of DNA damage, post-translational modifications such as phosphorylation and acetylation may also play a role in enhanced p53 levels^30,32^. In our study, increased expression of phosphorylated p53 was found after gimatecan treatment, which is consistent with this viewpoint.

The increased expression of p53 after CPT treatment results in cell cycle arrest^33–35^. Irinotecan may induce cell cycle arrest in different phases including the S-phase and G2/M-phase in testicular cancer^36^, colon cancer^37^, and non-small cell lung cancer^38^. Gimatecan induces S-phase arrest in bladder carcinoma, ovarian carcinoma, and melanoma^21,39^. In our study, both gimatecan and irinotecan induced intra-S-phase arrest in EC in vitro and in vivo. These findings are similar to previous studies, except for a small discrepancy that might be due to the inherent heterogeneity of different tumor types, because the multiple signals that regulate the cell cycle can be cell type-specific. We hypothesized that activation of p53 functioned as a transcriptional activator of some target genes including p21 WAF1. Activated p21 proteins interact with CDK2/cyclin A2 and inhibit binding of CDK2/cyclin A, resulting in S-phase arrest.

Activated p53 may induce not only cell cycle arrest but also apoptosis activation or cellular senescence^30,31^. Elevation of p53 induces Bax expression, downregulates the anti-apoptotic protein Bcl-2, and activates the caspase 3/7/9-dependent pathway, which is associated with the inhibition of tumor cell growth^31^. In our study, the expression of Bcl-2 protein significantly decreased, while cleaved-caspase 3 and cleaved-caspase 9 increased after gimatecan treatment, which indicates that gimatecan induced apoptosis. Cellular senescence was also evaluated in vitro after gimatecan treatment for 24 h, 48 h, 72 h, and 96 h using the senescence-associated beta galactosidase detection solution, and no senescence was observed.

Gimatecan retained stronger potency in antitumor efficacy compared to irinotecan in ESCC in this study. Similar results have also been reported in prostate carcinoma^40^ and neuroblastoma^41^, and the modified structure contributes to it a lot. Among a series of seven substituted camptothecins, gimatecan was developed with lipophilic modification at position C-7. The modification enhances stability of the drug–enzyme–DNA complex and lactone ring, and ensures rapid agent intake and stable drug interactions with intracellular targets. Furthermore, gimatecan has a favorable tissue distribution, with particular reference to the ability to cross the blood–brain barrier as a consequence of lipophilic modification, which is also supported by in vivo distribution studies in glioma preclinical models^42^. It is likely that the cytotoxic potency exhibited by gimatecan is the result of the combination of these features. In additional, oral administration is much more convenient than intravenous injection, which is the method used to administer irinotecan. Intestinal absorption is crucial for oral treatment, and could ensure a high drug concentration in the liver, which is a common site of metastasis for ESCC. In contrast to irinotecan and topotecan, gimatecan is not a substrate for breast cancer resistance protein (BCRP), an efflux pump for the multi-drug resistance P-glycoprotein^22^, so gimatecan is able to overcome cellular resistance in mitoxantrone-selected cell lines characterized by high levels of BCRP expression^43^.

In summary, our study demonstrates that by suppressing topoisomerase I, inducing S-phase cell cycle arrest and DNA damage, and promoting apoptosis, the new camptothecin analog gimatecan strongly inhibits ESCC growth both in vitro and in vivo. In addition, it shows improved antitumor efficacy compared to the current first-line agent, irinotecan, which indicates that this new oral medicine may serve as a better clinical option for ESCC-targeted therapy.

## Materials and methods

### Agents

Gimatecan (7-t-butoxyiminomethylcamptothecin, purity ≥ 99.9%) was synthesized and kindly provided by Zhaoke Pharmaceutical Ltd (Hefei, China)^22,42^. The chemical agent was dissolved in dimethylsulfoxide (DMSO) and stored at −80 °C. Irinotecan (purity ≥ 99.9%) was purchased from Jiangsu Hengrui Medicine Co., Ltd (Jiangsu, China), and was dissolved in sterile distilled water before use.

### Cell lines and cell viability assay

Esophageal squamous carcinoma cell lines, including EC-109, KYSE450, KYSE-140, KYSE-510, and TE-1, TE-10, were used in this study. Cells were maintained in RPMI 1640 media (Gibco-BRL, MD, USA) supplemented with 10% fetal bovine serum (FBS, Gibco) and 1% penicillin-streptomycin (Gibco) in a humidified incubator (37 °C) with 5% CO_2_.

Cells were seeded into 96-well plates at a density of 2–5 × 10^3^ cells/well overnight in complete medium, and then were treated the next day with gimatecan (0–1000 nM) or irinotecan (0–1 × 10^6^ nM). After 48 h incubation, cell viability was measured using a Cell Counting Kit-8 (CCK8) assay (Dojindo, Kumamoto, Japan) according to the manufacturer’s instructions. Absorbance was measured at 450 nm using a spectrophotometer. All experiments were repeated and read at least three times. IC50 was defined as the concentration of drug causing a 50% reduction in cell number compared to that of untreated controls. The concentration of 30 nM gimatecan or 10 × 10^3^ nM irinotecan was applied for in vitro experiments according to the IC50 values.

### DNA topoisomerase I activity assay

DNA strand breakage induced by topoisomerase I was evaluated by the conversion of the double-stranded supercoiled DNA to a relaxed form. Cells were treated with gimatecan or irinotecan in a dosage-dependent manner for 2 h, and a time-dependent manner at 30 nM gimatecan and 10 × 10^3^ irinotecan, and then nucleoli were isolated. Topoisomerase I activity was assessed in isolated nucleoli using an experimental kit (TopoGen Inc., Port Orange, FL) according to the manufacturer’s instructions. Briefly, a 20 µL reaction containing nucleoli extract protein was incubated with supercoiled DNA (100 ng, provided by the kit) in 1 × reaction buffer (10 mM Tris-HCl, pH 7.9, 1 mM EDTA, 1.5 M NaCl, 0.1 mM spermidine, 50% glycerol, 0.1% BSA) for 30 min at 37 °C and terminated using stop loading buffer (1% sarkosyl, 0.025% bromophenol blue, 5% glycerol). Then the samples were loaded on a 1% agarose gel and run in 1 × TAE (40 mM Tris-Acetate, 1 mM EDTA, pH 8.3) buffer for 80 V for 30 min. The gels were stained with 0.5 mg/mL ethidium bromide for 30 min and then destained in distilled water for 20 min. After that, the gels were photographed using a UV transilluminator (Bio-Rad, Hercules, CA).

### Flow cytometry

To analyze the changes in cell cycle progression, cell pellets were harvested after exposure to gimatecan or irinotecan for 0 h, 4 h, 8 h, and then fixed in 70% cold ethanol at 4 °C overnight. Then the fixed cells were stained with 50 μg/mL propidium iodide (BD Biosciences), and incubated at room temperature in darkness for 30 min. Flow cytometry was performed using a FACS Calibur system (BD Biosciences) and data were analyzed by ModFit 4.0 software (BD Biosciences).

For cell apoptosis analysis, cells were exposed to gimatecan or irinotecan for 24 h, 48 h, or 72 h, and then stained with Annexin V- phycoerythrin PE and 7-amino-actinomycin (7-AAD) (BD Biosciences, Erembodegem, Belgium) at room temperature in the dark for 15 min. The flow cytometric analyses were conducted within 30 min. Cell apoptotic data were analyzed using the FlowJo software (TreeStar, Inc., Ashland, OR).

### Antitumor activity studies

In vivo antitumor experiments were conducted using 6- to 8-week-old female NOD/SCID mice (Beijing Vital River Laboratory Animal Technology Co., Ltd.). The mice were maintained in laminar flow rooms, with constant temperature and humidity and free access to food and water. All animal experiments were performed according to the animal experimental guidelines of Peking University Cancer Hospital and followed internationally recognized Animal Research: Reporting of In Vivo Experiments guidelines. Tumor cell line xenografts were established by subcutaneous injection of 2 × 10^6^ cells from in vitro cultures. Patient-derived xenograft (PDX) models were established in our lab according to previous studies^44^. Tumors were measured with fine calipers twice a week. When tumors were ~150–200 mm^3^, mice were randomly assigned into the treatment group or the control group (5–6 per group). The mice in the control group were treated with saline containing 10% DMSO. The mice in the treatment group were given gimatecan (0.25 mg/kg, d1–d5/week, oral gavage) or irinotecan (40 mg/kg twice weekly, intraperitoneal injection) according to previous reports^45,46^. All of the animals were treated for 3 weeks. At the end of treatment, the tumor tissues of PDX models were harvested and kept at −80 °C.

Parameters related to tumor growth were used as previously reported^44^: Tumor volume = (Length × Width)^2^/2; Tumor growth inhibition (TGI) = Δ*T*/Δ*C* × 100%; (Δ*T* = tumor volume change in the treatment group on the final day of the study, Δ*C* = tumor volume change in the control group on the final day of the study).

### Western blots

Eca-109 and KYSE-450 cells were seeded in 6-well plates with complete medium. Twenty-fourth after planting, cells were treated with 30 nM gimatecan or 10 × 10^3^ nM irinotecan for 48 h and then harvested. The cell pellets and tumor tissues of xenografts were lysed and protein concentrations were measured. Protein samples were diluted to equal concentrations (10 µg/µL), and separated by 8–12% SDS-PAGE. After being transferred onto nitro-cellulose membranes (GE Healthcare, Piscataway, NJ), samples were incubated with corresponding primary antibodies in 5% BSA at 4 °C overnight, then washed and incubated in secondary antibodies at room temperature for 1 h. Antibodies in this study included topoisomerase I, Cyclin A2, (Santa Cruz, CA, USA), p-ATM (Ser1981), p-ATR (Ser428), p-BRCA1 (Ser1524), p-Chk1(Ser345), p-Chk2 (Thr68), p-H2AX (Ser139), p-p53 (Ser15), p-CDC25C (Ser216), p21(Waf1/Cip1), p53, CDK2, Bax, Bcl-2, Cleaved-Caspase 3, Cleaved-Caspase 9 (Cell Signaling Technology, Boston, MA, USA), and β-actin (Sigma-Aldrich, USA). Proteins were visualized using ECLplus Western Blotting Detection Reagents (GE Healthcare).

### Statistical analysis

Statistical analyses, including one-way analysis of variance and the two-tailed Student’s *t*-test, were performed using SPSS 21.0 software (Inc., Chicago, IL, USA). Data are expressed as the mean ± standard deviation in each case. *P* < 0.05 was considered statistically significant.

## Electronic supplementary material

Supplementary Figure legends

Supplementary Figure 1

Supplementary Figure 2

Supplementary Figure 3
