# Supplementary material for: A novel oral camptothecin analog, gimatecan, exhibits superior antitumor efficacy than irinotecan toward esophageal squamous cell carcinoma in vitro and in vivo
Source: Cell Death Dis. 2018 May 31;9(6):661. doi: 10.1038/s41419-018-0700-0 (PMC5981453; doi:10.1038/s41419-018-0700-0)
Supplement: Supplementary file 1 — Supplementary Figure legends [file 41419_2018_700_MOESM1_ESM.docx]

**Suppementary Figure legends**

**Supplement Fig 1 Higher dosage of irinotecan reduce topoisomerase I specific activity in ESCC**

**a.** Agent dilution relation assay: Eca-109 and KYSE-450 cell lines were exposed to serial dilutions of gimatecan and irinotecan for 2 h, and 20 µL of reaction containing nucleoli extract protein was incubated with supercoiled DNA for 30 min. Lane 1, supercoiled DNA only. Lane 2, Relaxed DNA that used as negative control. Lanes 3–8, supercoiled DNA, and nucleoli extract protein of cells treated with gimatecan (0–80 nM) or irinotecan (0–40 μM) for 2 h; **b.** Time-course experiment of topoisomerase I activity using a 0.2 µg reaction containing nucleoli extract protein per reaction. The extract protein was incubated with supercoiled DNA for 30 min at 37°C. Lane 1, supercoiled DNA only. Lane 2, Relaxed DNA that used as negative control. Lanes 3–7 (right) cells were treated with gimatecan (30 nM) or irinotecan (10 μ M) for 0.5 h to 4 h. Sc, supercoiled DNA; R, relaxed DNA.

**Supplement Fig 2 Higher dosage of irinotecan induces DNA damage in ESCC**

Eca-109 and KYSE-450 cell lines were exposed to 30nM gimatecan and 10 uM irinotecan for 48 h, and harvested at 70–80% confluence. Total protein was extracted from harvested cell lines or tumor tissues, and the expression of the following DNA damage-related proteins were assessed by Western blotting: p-ATM, p-ATR, p-BRCA1, p-H2AX, p-CHK1, p-CHK2, p53, and p-p53.

**Supplement Fig 3 Higher dosage of irinotecan induces S-phase arrest and apoptosis in ESCC**

**a**. Eca-109 and KYSE-450 cells were treated with 30nM gimatecan and 10μM irinotecan for 4 h or 8 h. Cell cycle progression was assessed after release using propidium iodide staining detected by fluorescence activated cell sorting. Sums of percentages of each cycle were also calculated in Eca-109 and KYSE-450. Results are representative of three independent experiments. **b.** 30nM gimatecan and 10μM irinotecan significantly induced cell apoptosis in Eca-109 and KYSE-450 cells, according to flow cytometry assays. Cells were treated with gimatecan and irinotecan at the indicated dose for 72 h and stained with Annexin V-PE/7-AAD. Sums of percentages of early apoptosis (Q3) and late apoptosis (Q2) were calculated as total apoptosis ratios. Results are representative of three independent experiments.  **c.** The expressions of proteins related to the cell cycle and apoptosis were assessed by Western blotting *in vitro*. Eca-109 and KYSE-450 cell lines were exposed to 30nM gimatecan and 10μM irinotecan for 48 h, and harvested at 70–80% confluence. Cell cycle-related proteins, such as Cyclin A, CDK2, and p21, and Pro- and anti-apoptotic proteins including Bax, Bcl-2, cleaved-caspase 3, and cleaved-caspase 9 were assessed by Western blot. Data represent the mean ± SD of three replicate assays. * p<0.05, ** p<0.01, *** p<0.001
