# Supplementary figures and images for: A novel oral camptothecin analog, gimatecan, exhibits superior antitumor efficacy than irinotecan toward esophageal squamous cell carcinoma in vitro and in vivo
Source: Cell Death Dis. 2018 May 31;9(6):661. doi: 10.1038/s41419-018-0700-0 (PMC5981453; doi:10.1038/s41419-018-0700-0)

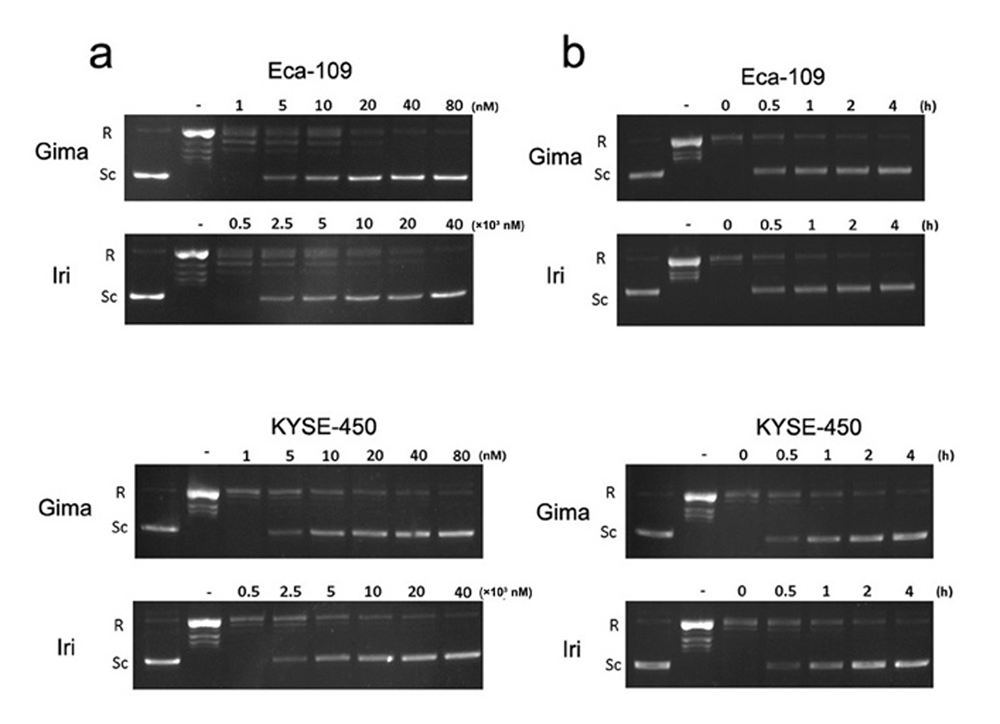

Supplement: Supplementary file 2 — Supplementary Figure 1 [file 41419_2018_700_MOESM2_ESM.tif]

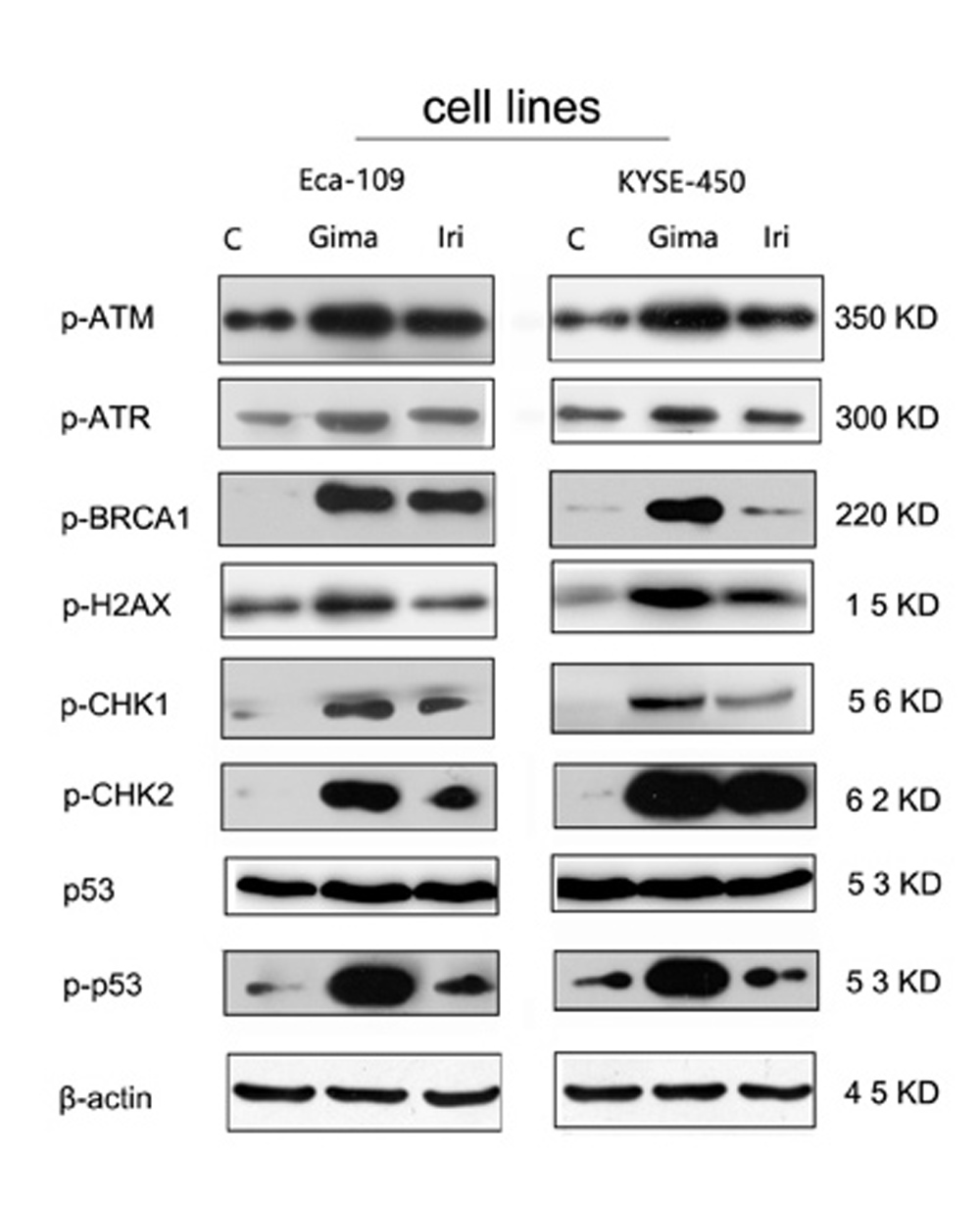

Supplement: Supplementary file 3 — Supplementary Figure 2 [file 41419_2018_700_MOESM3_ESM.jpg]

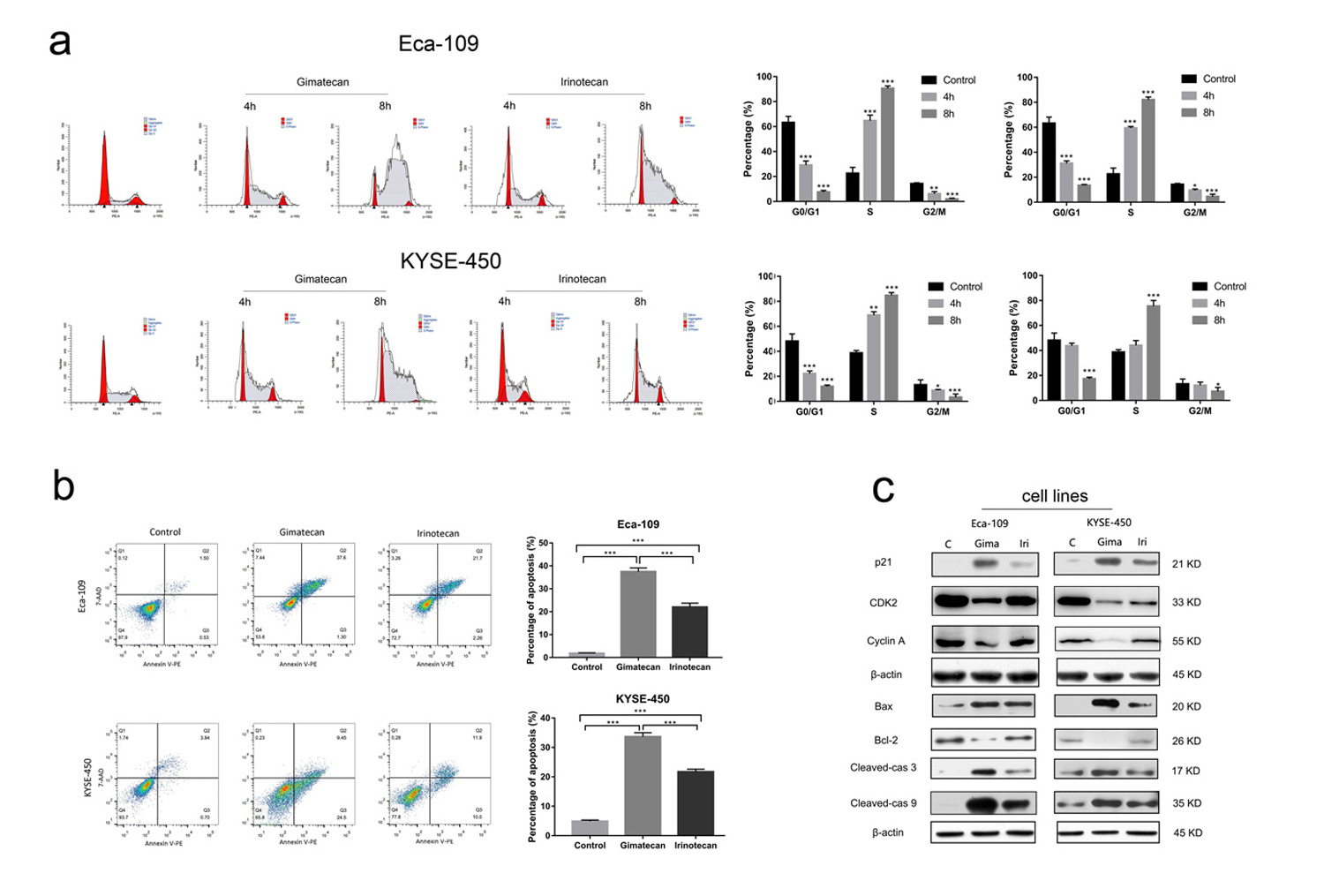

Supplement: Supplementary file 4 — Supplementary Figure 3 [file 41419_2018_700_MOESM4_ESM.jpg]
